# Outcomes and potential surrogate markers for future clinical trials of non‐alcoholic steatohepatitis cirrhosis

**DOI:** 10.1111/liv.15013

**Published:** 2021-07-21

**Authors:** Jesús Rivera‐Esteban, Angelo Armandi, Salvador Augustin, Elisabetta Bugianesi

**Affiliations:** ^1^ Liver Unit Department of Internal Medicine Vall d’Hebron Hospital Universitari Vall d’Hebron Institut de Recerca (VHIR), Vall d’Hebron Barcelona Hospital Campus Barcelona Spain; ^2^ Universitat Autònoma de Barcelona Bellaterra Spain; ^3^ Department of Medical Sciences Division of Gastroenterology and Hepatology A.O. Città della Salute e della Scienza di Torino University of Turin Turin Italy; ^4^ Centro de Investigación Biomédica en Red de Enfermedades Hepáticas y Digestivas (CIBERehd) Instituto de Salud Carlos III Madrid Spain

**Keywords:** biomarkers, clinical trials, liver cirrhosis, metabolic associated fatty liver disease (MAFLD), portal hypertension, portal pressure

## Abstract

Non‐alcoholic steatohepatitis has emerged as a major public health problem, and the burden of non‐alcoholic steatohepatitis cirrhosis is projected to increase by 64%‐156% by 2030. The threat is aggravated by the fact that are currently no approved drugs for the treatment of non‐alcoholic steatohepatitis. In this paper, we review the main challenges to drug development in patients with non‐alcoholic steatohepatitis cirrhosis, and describe the opportunities brought by the advances in the understanding of the clinical and pathophysiological nuances of cirrhosis. The design of therapeutic regimens for non‐alcoholic steatohepatitis cirrhosis will vary according to the specific cirrhosis substage (compensated vs decompensated), and the specific mechanistic basis of therapy, targeted either at improving aetiology‐specific pathways and/or at more general aetiology‐agnostic processes. The understanding of the probabilistic expectations for the whole range of potential outcomes, rooted at different mechanistic drivers at each specific substage, will be essential in order to choose adequate estimands and therapeutic strategies for clinical trials and individual patients with non‐alcoholic steatohepatitis cirrhosis. Finally, we provide a summary of the main pitfalls and uncertainties in the design of clinical trials for non‐alcoholic steatohepatitis cirrhosis and discuss potential biomarkers for use in trials and practice for these patients.

**Abbreviations**
ACLF: acute‐on‐chronic liver failure
CC: cryptogenic cirrhosis
CD: clinical decompensation
CSPH: clinically significant portal hypertension
CVD: cardiovascular disease
ELF: enhanced liver fibrosis
FDA: Food and Drug Administration
HCC: hepatocellular carcinoma
HE: hepatic encephalopathy
HVPG: hepatic venous pressure gradient
Hyperdynamic circ. Sd.: hyperdynamic circulatory syndrome
IHVR: intrahepatic vascular resistance
MELD: model for end‐stage liver disease
NAFLD: non‐alcoholic fatty liver disease
NASH: non‐alcoholic steatohepatitis
OLT: orthotopic liver transplantation
PBF: portal blood flow
PH: portal hypertension
PRO: patient reported outcome
RCTs: randomized controlled trials
T2DM: type 2 diabetes mellitus

**Key points**

- Non‐alcoholic steatohepatitis (NASH) represents a major public health problem, with the burden of NASH cirrhosis projected to increase 64%‐156% by 2030.
- There are no Food and Drug Administration‐approved drugs for NASH cirrhosis, and clinical outcomes are the only recommended endpoints for market approval.
- Therapeutic regimens will vary according to cirrhosis stage (compensated vs decompensated) and the mechanistic basis of therapy (aetiology specific vs symptomatic).
- A clear definition of NASH cirrhosis, understanding of liver disease biology and detailed patient risk stratification are required for future clinical trials.
- Non‐invasive tests, eg enhanced liver fibrosis and liver stiffness, are promising biomarkers.

## 1 THE BURDEN OF CIRRHOSIS DUE TO NON‐ALCOHOLIC STEATOHEPATITIS (NASH)

Non‐alcoholic fatty liver disease (NAFLD) has emerged as a major public health threat, and the burden of end‐stage liver disease due to NAFLD is projected to increase from 64% to 156% by 2030.^1^ All longitudinal studies conducted on biopsy‐proven NAFLD cohorts over the past 2 decades have highlighted the number of patients with cirrhosis. The prevalence of cirrhosis is currently estimated to range from 0.6% to 65%, with a median value of 9% (Table 1).^2^, ^3^, ^4^, ^5^, ^6^, ^7^, ^8^, ^9^, ^10^, ^11^, ^12^, ^13^, ^14^, ^15^, ^16^ Overall mortality in patients with NAFLD is predicted to be approximately 20%, while liver‐related mortality is approximately 4% across all stages of fibrosis, showing a stepwise progression from mild to severe fibrosis.^17^, ^18^, ^19^

**TABLE 1:** Prevalence of cirrhosis, and overall and liver‐related mortality in longitudinal studies of biopsy‐proven NAFLD cohorts^2^, ^3^, ^4^, ^5^, ^6^, ^7^, ^8^, ^9^, ^10^, ^11^, ^12^, ^13^, ^14^, ^15^, ^16^

Study	Year of publication	Number of subjects	Cirrhosis prevalence (%)	All‐cause mortality (%)	Liver‐related mortality (%)	Follow up (years)
Matteoni et al^2^	1999	132	20.4	48.9	9.1	8.3
Dam‐Larsen et al^3^	2004	109	0.6	24.8	0.9	16.7
Adams et al^4^	2005	420	5^a^	12.6	1.7	7.6
Ekstedt et al^5^	2006	88	7.9	20.1	2.8	13.7
Sanyal et al^6^	2006	152	100^a^	19	4.6	10
Soderberg et al^7^	2010	118	9	40	6.7	28
Bhala et al^8^	2010	247	54	13.4	5.6	7.4
Younossi et al^9^	2011	210	10.5	26.6	8.5	12.1
Sebastiani et al^10^	2015	148	14.9	6	2	5
Ekstedt et al^11^	2015	224	1.8	42.4	4	26.4
Angulo et al^12^	2015	619	2.9	31.1	4.2	12.6
Seko et al^13^	2015	312	8	2.6	0.3	4.8
Leung et al^14^	2016	307	15.3	2.6	0.3	4.1
Hagström et al^15^	2017	646	3.1	33.1	7.9	20
Vilar‐Gomez et al^16^	2018	458	65.2	8	6.7	5.5
Median values			9	20.1	4.2	

Abbreviation: NAFLD, non‐alcoholic fatty liver disease.

^a^ Diagnosed either by biopsy or clinical/imaging assessment.

By 2030, cirrhosis and end‐stage liver disease related to NASH are projected to increase worldwide.^1^ Models of future disease burden for incident decompensated cirrhosis have shown the highest percentage in France (164%) followed by the USA (150%).^1^ In addition, projections from one Canadian study showed that cirrhosis due to NASH will increase in all birth cohorts by 2040, accounting for 73% of all new diagnoses of cirrhosis.^20^

As shown in Table 2, the median proportion of cirrhotic patients with liver decompensation in longitudinal studies of biopsy‐proven NAFLD cohorts is 13.4%, ranging from 3% to 45% across the different cohorts.^4^, ^5^, ^6^, ^8^, ^10^, ^12^, ^13^, ^14^, ^15^, ^16^ Clinical events related to portal hypertension are the most common findings: ascites (median 8.2%, min–max: 0.4%‐70%), variceal bleeding (median 8.6%, min–max: 0%‐66.4%) and hepatic encephalopathy (median 4%, min–max: 0%‐31.6%). Accordingly, the development of liver decompensation constitutes a healthcare burden that requires high resource utilisation, and is related to inpatient and short‐term mortality.^21^ Onset of hepatocellular carcinoma (HCC) represents another relevant clinical outcome, as well as occurrence of liver transplantation (median 0.5%, min–max: 0%‐34.2%), that underlines the progressiveness of NAFLD. Otherwise, cardiovascular disease (CVD) involves a low rate of events in cirrhotic patients compared with liver‐related outcomes and to the relative weight of morbimortality due to CVD in pre‐cirrothic stages.^16^

**TABLE 2:** Proportions of cirrhotic patients with overt decompensation and most frequently reported liver‐related events in longitudinal studies of biopsy‐proven NAFLD cohorts

Study	Liver decompensation (%)	Ascites (%)	Variceal haemorrhage (%)	HCC (%)	HE (%)	OLT (%)
Adams et al^4^	3.1	2	1	0.5	2	0.2
Ekstedt et al 2006^5^	5.7	4.5	1.1	2.3	0	2.3
Sanyal et al^6^	45	42.8	66.4	1.2	31.6	34.2
Bhala et al^8^	19.4	7.7	10.5	2.4	7.7	NA
Sebastiani et al^10^	16.2	8.7	6.7	0.7	0	1.3
Angulo et al^12^	13.4	34.6	46	11.5	23.1	0.5
Seko et al^13^	NA	NA	NA	1.9	NA	0
Leung et al^14^	1.6	0.4	0	0.9	0.4	0
Hagström et al^15^	11.8	NA	NA	1.9	NA	0
Vilar Gomez et al^16^	19	70	24	9	6	8
Median values	13.4	8.2	8.6	1.9	4	0.5

Abbreviations: HCC, hepatocellular carcinoma; HE, hepatic encephalopathy; NA, not applicable; OLT, orthotopic liver transplantation.

One recent study in Wales^22^ conducted in nearly 70 000 individuals affected by cirrhosis has shown that the incidence of NAFLD has increased 10‐fold over the past 10 years and has become the predominant cause of liver damage. As compared with other aetiologies, the course of NASH‐related liver disease appears to be milder, with a smaller proportion of decompensated patients (8% of patients with NASH cirrhosis, vs 25% of patients with alcohol‐related cirrhosis). However, clinical outcomes extracted from current longitudinal studies need to be related to the rapidly increasing burden of NASH cirrhosis, which will eventually cause a considerable increase in liver‐related events and mortality.

## 2 CURRENTLY APPROVED ENDPOINTS IN NASH CIRRHOSIS TRIALS

There are currently no Food and Drug Administration (FDA)‐approved drugs for compensated NASH cirrhosis. Patients with cirrhosis are the most likely to develop hard outcomes (eg death, HCC, liver transplantation). Current trials in patients with cirrhosis capture outcomes related to cirrhosis, such as hepatic encephalopathy, ascites and variceal haemorrhage, as well as the laboratory components of the Child–Pugh score. Improvement in histological fibrosis stage or hepatic collagen content have been also used as primary endpoints of outcome events in trials focused on NASH cirrhosis. However, the reversal of cirrhosis to lesser degrees of fibrosis is an ambitious goal, and the relationship between histological changes in cirrhosis and clinical outcomes has not been characterised. Improvements in fibrosis stage may reflect either true regression or sampling bias.

Currently, the only endpoints recommended by the FDA to support marketing approval in compensated NASH cirrhosis are clinical outcomes.^23^ According to the FDA, Phase III trials in patients with compensated NASH cirrhosis should evaluate the effect of the investigational drug relative to placebo on the composite endpoint of time from randomisation to the first of any one of the following outcome events:

1. Complication of ascites including any of the following: spontaneous bacterial peritonitis, diuretic‐resistant ascites (refractory ascites), hepato‐pleural effusion.
2. Variceal haemorrhage.
3. Hepatic encephalopathy.
4. Worsening model for end‐stage liver disease (MELD) score to greater than or equal to 15 (this endpoint approximates listing for liver transplant).
5. Liver transplantation.
6. Death from any cause.

## 3 WHICH PATIENTS WITH NASH CIRRHOSIS ARE ELIGIBLE FOR PHARMACOLOGICAL THERAPY AND HOW TO SELECT THEM: THE DIFFERENT STAGES OF NASH CIRRHOSIS

The structure and focus of therapeutic regimens for NASH cirrhosis will vary according to 2 main factors: (i) the specific substage of cirrhosis at which the therapeutic strategy is directed, and (ii) the specific mechanistic basis of therapy–either targeted at improving aetiology‐specific pathways (such as the metabolic derangements typical of NASH) or at more general aetiology‐agnostic processes (such as the intrahepatic or extrahepatic vascular dysregulation common to all forms of portal hypertension).^24^, ^25^, ^26^

The classical concept of cirrhosis is histological in nature, and has been traditionally regarded as a static, non‐reversible last stage for all forms of progressive liver diseases.^24^ However, both the success of antiviral therapies in cirrhosis, as well as the integration of clinical and haemodynamic knowledge generated in the past 2 decades, have helped to develop a more comprehensive, dynamic and nuanced view of this last phase of chronic liver disease.^24^ Nowadays, there are at least 4 distinct, well‐differentiated substages of cirrhosis, classified according to the mechanisms driving progression and potential for regression of disease at each stage and the expected probabilities of a different range of outcomes.^24^, ^26^ Table 3 summarises a schematic view of these 4 substages and the potential implications for the treatment of patients with NASH cirrhosis.

**TABLE 3:** The different stages of NASH cirrhosis: Clinical features, mechanistic drivers and ideal therapeutic goals and endpoints

	Compensated NASH cirrhosis		Decompensated NASH cirrhosis	
	Early compensated	Late compensated (compensated CSPH)	Early decompensated	Further decompensated
Clinical features	Unspecific symptoms	Unspecific symptoms (± varices)	Few/mild decompensating events	Recurrent/refractory decompensating events ACLF (multiorgan failure)
Biological features				
Histology	Scar, X‐linking (thin septa)	Acellular scar, nodules (thick septa)	Insoluble scar	Insoluble scar
Haemodynamic (HVPG)	5‐10 mm Hg (mild PH)	>10 mm Hg (CSPH)	>12 mm Hg	>16 mm Hg
Mechanistic drivers				
Primary	NASH mechanisms (metabolic/ inflammation/ fibrosis)	NASH mechanisms (fibrosis) Increased IHVR	Increased portal pressure (PBF+IHVR) Hyperdynamic circ. Sd.	Failed hyperdynamic compensation Decreased liver function
Secondary	Increased IHVR	Increased PBF Collaterals development	Decreased liver function Increased inflammation Increased bacterial translocation Hypercoagulability	Increased inflammation Increased bacterial translocation Hypercoagulability Increased portal pressure
Therapeutic goals/endpoints				
Primary	Improvement in NASH histology (NASH resolution and/or fibrosis regression?)	Prevention of liver clinical events (CD)	Transplant‐free survival Overall survival	Overall survival PROs
Secondary	Prevention of liver +non‐liver clinical events Resolution of PH Decreased progression to CSPH New fibrosis biomarkers?	Improvement in NASH histology Prevention of non‐liver clinical events Decrease in HVPG (>20% and/or <10 mm Hg) New fibrosis/liver function biomarkers?	Recurrence/control of CD events Mechanistic/proof‐of‐concept: specific to type of CD Changes in MELD/Child‐Pugh scores PROs	Recurrence/control of CD events Mechanistic/proof‐of‐concept: specific to type of CD Post‐transplant outcomes

Abbreviations: ACLF, acute‐on‐chronic liver failure; CD, clinical decompensation; CSPH, clinically significant portal hypertension; HVPG, hepatic venous pressure gradient; Hyperdynamic circ. Sd., hyperdynamic circulatory syndrome; IHVR, intrahepatic vascular resistance; MELD, model for end‐stage liver disease; PBF, portal blood flow; PH, portal hypertension; PRO, patient reported outcome.

From a clinical standpoint, the occurrence of a first clinical decompensation (CD) indicates a dramatic worsening of prognosis in patients with cirrhosis and enables division into 2 main stages: compensated and decompensated cirrhosis.^27^ Indeed, the expected median survival of a patient with compensated cirrhosis is 10‐12 years, compared with the 1.5‐2 years that could be expected after a patient's first decompensating event^28^, ^29^ (of note, these rates are drawn from studies including patients with all aetiologies of cirrhosis–the impact on survival of the transition from compensated to decompensated stage has not been reported specifically for NASH‐).

The compensated stage is the longest phase and can go undiagnosed given its asymptomatic nature. Nonetheless, early identification of patients at this stage would be of paramount importance, since this is precisely the phases at which therapies might be still able to prevent the transition to the decompensated stage, or even lead to a regression to pre‐cirrhotic stages. Within the compensated stage, the presence and degree of portal hypertension is a key predictor of outcome. By integrating haemodynamic data, further distinction of prognostic relevance can be made into an early compensated stage and a late compensated stage.^24^, ^26^, ^27^, ^30^ The early compensated stage is characterised by a mild degree of portal hypertension (defined by an hepatic pressure venous gradient (HVPG) <10 mm Hg), and a low risk of CD (<10% at 4 years).^31^, ^32^ The late compensated stage is characterised by the presence of clinically significant portal hypertension (CSPH), defined by an HVPG ≥10 mm Hg, the threshold above which CD is up to 4 times more likely to occur.^31^, ^32^

The distinction between the early compensated stage and CSPH is not merely prognostic but also carries pathophysiological and therapeutic implications. In the early compensated stage, the main drivers of progression and outcomes are aetiology‐specific.^25^, ^26^ There seem to be intrahepatic vascular adaptations, both structural and functional–the latter being characterised by a moderate increase in intrahepatic vascular resistance (IHVR)–all of which translates into mild portal hypertension.^30^, ^33^ Thus, within this stage, therapeutic efforts should be directed towards arresting the driving mechanism of NASH. The underlying assumption (extrapolated from the lessons learnt from antiviral therapies^34^, ^35^, ^36^, ^37^), is that the improvement of those aetiology‐specific mechanisms will carry over indirectly into the arrest (or even regression) of fibrosis and the functional improvement of IHVR, leading to a decrease of portal pressure and the risk of CD. Within this clinical and pathophysiological framework, the choice of outcomes in this specific early stage should focus on histological improvement of NASH features and fibrosis as the main endpoint (as in current Phase III trials). Progression to CSPH and/or resolution of portal hypertension could be considered as secondary outcomes or for earlier Phase II trials.^25^ In order to assess the impact of hard clinical endpoints in this early NASH cirrhosis population, large sample sizes and a longer duration of follow up are likely to be required, as seen in current Phase III/IV trials.^32^, ^38^

Once the critical CSPH threshold is reached, the risk of CD increases considerably,^27^, ^31^, ^32^ and therefore the prevention of CD should become the main therapeutic goal and the ideal endpoint for trials.^25^ During this late compensated stage, structural intrahepatic changes are characterised by the presence of thicker fibrotic septa, thus making the regression of fibrosis unlikely.^24^ Also, extrahepatic adaptations start to develop, with an increase in portal blood flow, the development of collateral circulation and systemic adaptations leading to the initial phases of a mild hyperdynamic circulatory state.^39^, ^40^ Once these adaptations appear, splanchnic vasoconstrictors start to become effective at halting the progression of portal hypertension and reducing the risk of decompensation.^40^, ^41^ Extrapolating from experience with antiviral therapies, it can be assumed that for NASH cirrhosis, there might also be a ‘point of no return’ somewhere after CSPH is established, for which the effects of aetiology‐specific therapies are no longer able to reverse CSPH and eliminate the risk of CD without concomitant medication.^34^, ^35^, ^36^, ^37^ Under these pathophysiological assumptions, therapeutic efforts in this late compensated phase should aim to target the same aetiology‐specific mechanistic pathways as well as both intrahepatic and extrahepatic vascular tone. As a matter of fact, in patients already harbouring oesophageal varices, the use of non‐selective beta‐blockers is the standard of care^25^ and cannot be avoided as a comparator for trials.^26^ The transition of HVPG values to <10 mm Hg could be considered as a potentially relevant surrogate endpoint in these patients,^25^, ^26^ but histological changes in NASH and fibrosis become less likely^32^ and are thus not ideal endpoints.

Finally, the occurrence of CD signals that intrahepatic and extrahepatic structural and functional adaptations are insufficient and therefore that the patient has progressed to NASH cirrhosis.^24^, ^30^ The risk of further decompensation, progression to end‐stage liver disease and death increases exponentially as decompensating events accumulate.^26^, ^28^ From a pathophysiological perspective, there is a progression of the hyperdynamic circulatory state, peaking in patients with refractory ascites, for whom there is a relative decrease in the cardiac index that leads to a further decompensated stage.^24^, ^26^ Mounting evidence also points to a deleterious hypercoagulable state in decompensated cirrhosis, especially affecting the micro‐circulation.^42^ All of these changes, along with the decrease in synthetic liver function, lead to an increase in gut bacterial translocation and systemic inflammation, which are key drivers of the decompensating inertia of this stage, eventually leading to a final high‐mortality stage characterised by multiorgan failure.^24^, ^27^ The role of aetiology‐specific therapies is likely secondary in these later stages and, for the specific case of NASH, might even be associated with deleterious effects, especially with weight loss‐directed approaches, which could lead to worsening of patients’ nutritional and performance status and an increased risk of complications and death.^43^, ^44^ Aetiology‐agnostic approaches in this phase might be more generally directed to the modification of intra and extrahepatic vascular tone dysregulation or can be more specifically targeted to the specific complications (control of fluid retention for ascites, nitrogen metabolism for hepatic encephalopathy [HE], etc). Therapeutic efforts to control infection and systemic inflammation, and even to counterbalance the described hypercoagulable state, should also have a role in this stage.

Considering the poor prognosis in the decompensated stage, transplant‐free survival is recommended as the primary endpoint for Phase III trials.^25^, ^45^ Either surrogate or secondary endpoints for Phase III trials (such as development of multiorgan involvement as in acute‐on‐chronic liver failure), or primary endpoints for Phase II trials focusing on specific decompensating events (recurrence of variceal haemorrhage, hepatic encephalopathy, control of ascites, etc), should be chosen according to the mechanism of action of the drug under investigation and the specific targeted patient subpopulation. Inclusion of patient reported outcomes should become standard practice in this symptomatic, late stage of NASH cirrhosis. Three excellent recent reviews provide a comprehensive view of the different tools and approaches for reporting quality of life in patients with advanced liver disease.^46^, ^47^, ^48^

In summary, the understanding of the probabilistic expectations for the whole range of potential outcomes, rooted at different mechanistic drivers at each specific substage of NASH cirrhosis, is essential in order to choose adequate estimands and therapeutic strategies for clinical trials, and tailor therapy to individual patients with NASH cirrhosis.

## 4 PITFALLS AND UNCERTAINTIES IN CLINICAL TRIALS FOR NASH CIRRHOSIS

### 4.1 Definition of NASH cirrhosis

An accurate case definition of NASH cirrhosis is critical for enrolling appropriate patients into clinical trials, but currently there are no published criteria for defining NASH cirrhosis. NASH is a histological diagnosis, but in clinical practice, the term NASH cirrhosis has been increasingly used by physicians to address the forms of end‐stage liver diseases in the presence of known risk factors for metabolic derangement, mainly obesity and type 2 diabetes mellitus. However, the progression of NASH is associated with a loss of intrahepatic fat and diminished inflammatory activity.^49^ Therefore, in the absence of a prior diagnosis of NASH, end‐stage liver histology cannot be linked with a specific aetiology, and the term NASH cirrhosis remains hypothetical. In this line, the FDA requires fulfilment of full NASH histological criteria for enrolment in phase 3 trials in the F4 population. This certainly represents a significant hurdle for recruitment, since aproximatelhy, half of F4 patients do not show full NASH features,^49^ which explain in part the high screen failure rate common in NASH trials.

The presence of a suggestive medical history and/or hallmarks of metabolic syndrome may help to characterise patients with true NASH cirrhosis, with respect to those with unexplained (‘cryptogenic’) cirrhosis (CC).^50^ Data suggest that patients with NASH cirrhosis and CC form different parts of the same spectrum of chronic liver disease that originates in the setting of metabolic abnormalities, as these patients have similar metabolic and clinical features, but are not currently enrolled in clinical trials because a threshold of steatosis (>5%) is required as an inclusion criterion. In a recent study among CC patients with <5% fat at baseline biopsy who had another biopsy during follow‐up, 40.4% had >5% fat on their subsequent biopsy, and, therefore, were no longer ‘cryptogenic’. Furthermore, patients with CC seem to have a more aggressive disease than those with NASH cirrhosis, as indicated by greater hepatic collagen content and α‐SMA expression on biopsy, higher serum fibrosis markers and MELD scores and a greater risk of liver‐related clinical events during follow‐up.^51^

The recent proposal to substitute the term NASH by Metabolic (dysfunction) Associated Fatty Liver Disease (MAFLD)^52^ stems in part from these challenges with the disease definition. The new term brings the focus to the metabolic substrate at the mechanistic and clinical basis of the disease. For the particular of NASH cirrhosis, this recognition may help at clarifying in part the conundrum of CC described above, since most patients with CC share the same metabolic substrate of NASH and have overlapping clinical progression. The new term also provides an enabling framework from where to reinterpret the coexistence in real practice of metabolic and alcoholic liver disease, with the recognition that the two conditions are not mutually exclusive and can coexist together. Also, the removal of specific thresholds of alcohol intake from the definition, forces the revision of the assumption that slight to moderate alcohol intake have no deleterious hepatic effects, which is implicit in the classical definition of NASH. This is also relevant in patients with NASH cirrhosis, in whom the potential impact of alcohol in the progression of the disease becomes especially critical.

Nonetheless, adoption of the new term is still a matter of debate, and proof of it is that the whole MAFLD concept has not been applied in clinical trials yet, where precise and well‐characterised criteria are still needed to avoid bias and confounders and for regulatory reassurance. In this regard, the Liver Forum recently developed a consensus case definition of NASH‐related cirrhosis for inclusion in clinical trials that may be qualitatively categorised according to the degree of certainty of NASH as the cause of cirrhosis: definitive, probable and possible.^53^ When histological evidence is absent, the presence of concomitant metabolic risk factors strengthens the likelihood that NASH is the cause of cirrhosis (Box 1).

BOX 1 **NASH Cirrhosis: Liver Forum consensus definitions for clinical trials^48^**

**Table:** 

1. Definite NASH cirrhosis	1a. Patients with current liver biopsy showing cirrhosis with steatohepatitis. 1b. Patients with a previous biopsy showing steatohepatitis, but now with evidence of cirrhosis, either by clinical history or current features, imaging, non‐invasive tests or biopsy. 1c. Patients with a current biopsy showing cirrhosis^a^ with steatosis (but no findings of active steatohepatitis) together with at least two coexisting or historical metabolic comorbidities including obesity and/or T2DM to corroborate a diagnosis of NASH as the cause of cirrhosis.
2. Probable NASH cirrhosis	2a. Patients with a previous biopsy with steatosis but not steatohepatitis, and current cirrhosis^a^. At least two coexisting or historical metabolic comorbidities including obesity and/or T2DM to support NASH as an underlying cause of cirrhosis. 2b. Patients with cirrhosis^a^ with current or previous imaging showing evidence of steatosis. At least two coexisting or historical metabolic comorbidities including obesity and/or T2DM to support NASH as an underlying cause of cirrhosis. 2c. Patients with ‘cryptogenic cirrhosis’ without current or previous evidence of steatosis by imaging or steatosis/steatohepatitis by histology. At least two coexisting or historical metabolic comorbidities including obesity and/or T2DM to support NASH as an underlying cause of cirrhosis.
3. Possible NASH cirrhosis	3a. Patients with ‘cryptogenic cirrhosis’ without current or previous evidence of steatosis by imaging or steatosis/steatohepatitis by histology. At least one coexisting or historical metabolic comorbidity including obesity and/or T2DM.

^a^ Either by a clinical history or current features, imaging, non‐invasive tests or biopsy.

Abbreviations: NASH: non‐alcoholic steatohepatitis; T2DM: type 2 diabetes mellitus.

### 4.2 Biology of liver disease in cirrhosis

The drugs currently in development for NASH can be divided into 3 broad categories: metabolic, anti‐inflammatory and antifibrotic. Favourable metabolic effects from a drug are desirable in patients with NASH, but to what extent the partial correction of these metabolic abnormalities leads to a more favourable outcome in cirrhosis is unknown. The biology of clinical decompensation in patients with previously compensated cirrhosis is not fully understood. For example, diabetes arising in liver cirrhosis (so‐called hepatogenous diabetes) has a profound impact on the pathology and natural history of the liver disease.^54^ Although peripheral insulin resistance and impairment of the hepatocellular function are two potential major causes, the beta‐cell capacity plays a critical role. Recent evidence that the failing liver exerts an independent ‘toxic’ effect on beta‐cells suggests that individuals with hepatogenous diabetes might benefit from interventions aimed at improving beta‐cell function, such as thiazolidinediones and incretins.^55^ On the other hand, cirrhosis is a condition of intense muscle protein wasting leading to sarcopenia even in individuals affected by obesity (so‐called sarcopenic obesity).^43^ Malnutrition and sarcopenia are associated with higher complication rates in patients with cirrhosis.^43^ Moreover, they are associated with increased mortality in hospitalised patients with cirrhosis and those waiting for liver transplantation.^43^ In this setting, drugs promoting weight loss may have a detrimental impact on the progression of cirrhosis. Therefore, it remains to be determined whether treatment of the underlying steatohepatitis as opposed to the fibrosis will prevent clinical decompensation. These possibilities are currently under active investigation.

### 4.3 Risk stratification

As described above, cirrhosis represents a broader spectrum of disease compared with its usual histological classification (F4), and the main challenge is the stratification of NASH patients with compensated cirrhosis to identify strata for decompensation that represent a clinically meaningful outcome. The expected risk of each outcome changes markedly from stage to stage, and sample size and follow‐up times should vary accordingly. This nuanced view of the different stages of cirrhosis is often overlooked in the design of large clinical trials in NASH cirrhosis. From an operational point of view, stratification by direct or indirect evidence of CSPH (HPVG>10 mm Hg, esophageal varices at endoscopy or, hopefully in the near future, non‐invasive biomarkers of CSPH) and/or a decreased liver function (Child‐Pugh score ≥6 points) could be used to fine‐tune the assumptions around expected event rates informing sample size, trial duration, etc A more granular and detailed sub‐stratification of patients with NASH cirrhosis and the corresponding choice of outcomes is needed to stimulate therapeutic development in this population.

### 4.4 Placebo reponse and Hawthorne effect

Placebo response represents a major challenge in NASH clinical trials. Patients allocated to placebo arms in therapeutic trials show significant histologic, radiologic and biochemical responses^56^, ^57^ that must be taken into account as they can interfere with the overall trial design and an adequate interpretation of results. Hawthorne effect is particulary relevant in a lifestyle‐driven disease such as NASH, as subjects may conciosuly or unconsciously change their behaviour after enrolment, directly affecting NASH biology and outcomes.^58^ To control this effect adequately, trials should include a standardised approach to lifestyle interventions and an objective assessment of lifestyle, including physical activity, dietary and alcohol intakes questionnaires. In this regard, the Liver Forum has recently issued a comprehensive review and position paper on this important topic, with specific recommendations.^59^ Trial duration may also affect the impact of the Hawthorne effect. Long trail durations, as currently required for phase 3 trials in NASH cirrhosis, might mitigate the potential impact of a Hawthorne effect (since lifestyle modifications are harder to maintain in the long term), but this is yet to be proven. Other factors such as sample size or geographic location must be also considered when interpreting the results of placebo arms in RCTs.

## 5 SUGGESTED SURROGATES AND NEW BIOMARKERS

### 5.1 HVPG

As discussed above, the HVPG remains the most robust and accurate surrogate predictor of outcomes in patients with compensated and early decompensated cirrhosis of different aetiologies, including NASH.^25^, ^31^ In patients with cirrhosis of viral aetiology, improvement of HVPG after antiviral therapy translates into a clear improvement in outcomes.^34^, ^35^, ^36^, ^37^ In the case of NASH cirrhosis, observations from the simtuzumab programme suggest that reductions in HVPG (at least 20% and/or below 10 mm Hg) is associated with a significant decrease in the risk of clinical decompensation.^32^, ^38^ However, HVPG measurement is technically challenging, invasive and expensive, and is still regarded as logistically demanding for use in large clinical trials. Furthermore, in the case of earlier compensated stages (before CSPH), which are the focus of most current programmes in NASH cirrhosis, more studies are required to demonstrate that improvement in HVPG (due to reduced fibrotic remodelling of the liver or other intrahepatic structural or functional mechanisms) translates into improved clinical outcomes. On this basis, the most recent FDA guidance on clinical trials in NASH cirrhosis does not discuss HVPG as a valid endpoint for marketing approval in Phase III studies.^53^ However, in the case of earlier development (Phases II and IIb), HVPG remains an attractive surrogate endpoint in compensated NASH cirrhosis, due to the large numbers of patients and long duration of follow up that would be required in trials with a primary endpoint of clinical outcomes, and the strong association of HVPG with clinical outcomes in this setting.

### 5.2 Improved histological readings

In an attempt to mitigate the inter and intra‐observer variability in NASH histology readings discussed in the first section, improved machine‐driven methods to quantify basal amount of fibrosis and changes in time are being increasingly explored in the last few years. A recent paper evaluating a machine‐learning (ML) based‐approach with paired biopsies from 3 large NASH RCTs including patients with advanced fibrosis (STELLAR‐3, STELLAR‐4 and ATLAS)^60^ showed that these artificial intelligence‐driven techniques are sensitive and reliable, and represent promising approaches to correlate dynamic changes in fibrosis (even in the F4 stage) with clinical outcomes, although the number of events in those clinical trials was very small and further studies are required to increase the confidence in this novel and exciting techniques.

### 5.3 New biomarkers in NASH cirrhosis

A major challenge in drug development is to identify and validate surrogate markers that predict a reduction in progression to hard outcomes. Different exploratory efficacy endpoints have been studied in clinical trials in patients with NASH cirrhosis, including changes in liver biochemistry tests; non‐invasive tests (eg enhanced liver fibrosis [ELF], liver stiffness by transient elastography); markers of apoptosis and necrosis and other histological measures including hepatic collagen and fat content and α‐SMA expression.^32^, ^61^ The demonstration that ELF score and liver stiffness had the ability to identify those most likely to progress from F3 to cirrhosis, in addition to predicting which patients with cirrhosis at baseline were most likely to have a liver‐related event,^61^ was of particular interest. The authors defined a change in ELF score of 0.5 points as the value that correlated with clinical liver events. This value also correlated with other non‐invasive tests, liver biochemistry tests, glycaemic parameters, CK‐18 values, serum bile acid values and body weight, but not with histological features. These findings give hope that in the future, reliance on histological endpoints will be a historical concept for NASH clinical trials. Validation from such biomarkers will hopefully be a reality as clinical events accumulate in ongoing Phase III trial efforts.

Functional testing is a novel approach to assess the severity of liver disease and changes in actual liver function in the context of treatment trials, especially in patients with cirrhosis. The HepQuant test simultaneously measure clearance from portal and systemic circulation as well as portal‐systemic shunting.^62^ Preliminary data indicate that this assessment of liver impairment correlates with hard endpoints, but more extensive validation is needed. Another test is the methacetin breath test, which is being evaluated in several ongoing clinical trials for NASH,^63^, ^64^ and longitudinal data that correlate this assessment with histological changes may be forthcoming. How the HepQuant, methacetin breath test and other tests of specific metabolic functions of the liver compare with clinical outcomes remains to be determined.

## 6 CONCLUSIONS

NASH cirrhosis has emerged as a major healthcare problem and finding effective therapies for patients with NASH has become one of the main unmet clinical needs in hepatology. Drug development in this field will face important challenges, due to the time needed to gather hard outcomes and the unreliable nature of fibrosis as an endpoint. A deeper understanding of the clinical nuances of NASH cirrhosis, and the mechanistic processes driving disease progression at each substage, will hopefully guide therapeutic efforts and appropriate patient selection, informative biomarkers and clinically meaningful endpoints.

## CONFLICTS OF INTEREST

Jesús Rivera‐Esteban reports no conflicts of interest. Angelo Armandi reports no conflicts of interest. Salvador Augustin is now an employee of Boehringer Ingelheim but was not when the manuscript was written. Elisabetta Bugianesi reports advisory board activity for Boehringer Ingelheim, Bristol Myers Squibb, Gilead, Innova, Intercept, Novo Nordisk.

## RESEARCH ETHICS AND PATIENT CONSENT

Not applicable.
